# Rational attenuation of RNA viruses with zinc finger antiviral protein

**DOI:** 10.1038/s41564-022-01223-8

**Published:** 2022-09-08

**Authors:** Daniel Gonçalves-Carneiro, Emily Mastrocola, Xiao Lei, Justin DaSilva, Yoke Fun Chan, Paul D. Bieniasz

**Affiliations:** 1 Laboratory of Retrovirology, The Rockefeller University, New York, NY USA; 2 Department of Medical Microbiology, University of Malaya, Kuala Lumpur, Malaysia; 3 Howard Hughes Medical Institute, The Rockefeller University, New York, NY USA

**Keywords:** Live attenuated vaccines, Restriction factors

## Abstract

Attenuation of a virulent virus is a proven approach for generating vaccines but can be unpredictable. For example, synonymous recoding of viral genomes can attenuate replication but sometimes results in pleiotropic effects that confound rational vaccine design. To enable specific, conditional attenuation of viruses, we examined target RNA features that enable zinc finger antiviral protein (ZAP) function. ZAP recognized CpG dinucleotides and targeted CpG-rich RNAs for depletion, but RNA features such as CpG numbers, spacing and surrounding nucleotide composition that enable specific modulation by ZAP were undefined. Using synonymously mutated HIV-1 genomes, we defined several sequence features that govern ZAP sensitivity and enable stable attenuation. We applied rules derived from experiments with HIV-1 to engineer a mutant enterovirus A71 genome whose attenuation was stable and strictly ZAP-dependent, both in cell culture and in mice. The conditionally attenuated enterovirus A71 mutant elicited neutralizing antibodies that were protective against wild-type enterovirus A71 infection and disease in mice. ZAP sensitivity can thus be readily applied for the rational design of conditionally attenuated viral vaccines.

## Abstract (web summary)

Rational design of live-attenuated RNA viruses with potential as vaccines is enabled by identification of sequence rules for zinc finger antiviral protein.

## Main

The zinc finger antiviral protein (ZAP) inhibits the replication of a broad range of RNA and DNA viruses^1–3^ through the recognition of viral CpG-rich RNA^4^ and presents opportunities for the design of attenuated viral vaccines. Live-attenuated viral vaccines offer advantages over other vaccine approaches because they express a complete repertoire of viral proteins and so present the widest range of antigenic determinants to induce durable cellular and humoral responses without adjuvants^5^. However, rational methods for the generation of attenuated viruses are few, and most attenuated vaccines have been empirically produced. One approach used for virus attenuation is recoding of nucleic acid sequences by synonymous mutagenesis. Initial reports using this method replaced codons or codon pairs with counterparts found only rarely in the human genome, a process termed ‘deoptimization’^6–10^. However, deoptimization of a viral RNA genome can have pleiotropic effects on structure, stability and translation efficiency, conferring virus attenuation through multifactorial mechanisms that are not straightforward to predict^11^.

Attenuation by codon-pair deoptimization incidentally increases the frequency of two dinucleotides, CpG and UpA (TpA in DNA)^12^. CpG dinucleotides are severely underrepresented in vertebrate genomes, while TpA/UpA dinucleotides are underrepresented in organisms across the tree of life^13^. The underrepresentation of CpG in vertebrate genomes has created an opportunity for non-self RNA recognition that is exploited by ZAP proteins^4^. The ZAP N-terminal domain employs a highly selective binding pocket that can only accommodate a CpG dinucleotide in a single-stranded configuration^14,15^. While one ZAP molecule binds to one CpG dinucleotide, individual CpG dinucleotides have negligible effects on viral replication. Rather, it is the cumulative effect of multiple CpG dinucleotides that enables ZAP antiviral activity^4^. However, it is unknown how CpG dinucleotide numbers, juxtaposition and underlying sequence context affect ZAP recognition of viral RNA. Moreover, even though CpG dinucleotides confer ZAP sensitivity, introduction of CpG dinucleotides in an unguided manner can have pleiotropic effects on viral replication through ZAP-independent mechanisms^16^.

Viral genome recoding without consideration of the mechanism(s) of attenuation may produce viruses with reduced immunogenicity, which is clearly an undesirable property for any vaccine^17^. Since ZAP–RNA interactions may be immunostimulatory^18^, optimal recoding strategies would maximize ZAP binding and specify ZAP recognition as the attenuating mechanism. However, so far, delineation of sequence features that could be employed to achieve this goal has not been reported.

Using HIV-1 as a model system, we define how CpG dinucleotide number, spacing and surrounding sequence affect ZAP sensitivity. We then apply these parameters to design a mutant picornavirus genome with precise and stable modifications that function as an effective live-attenuated vaccine whose replication is specifically inhibited by ZAP in cell culture and in vivo.

## Results

### CpG dinucleotides and HIV-1 replication

**Fig. 1:**
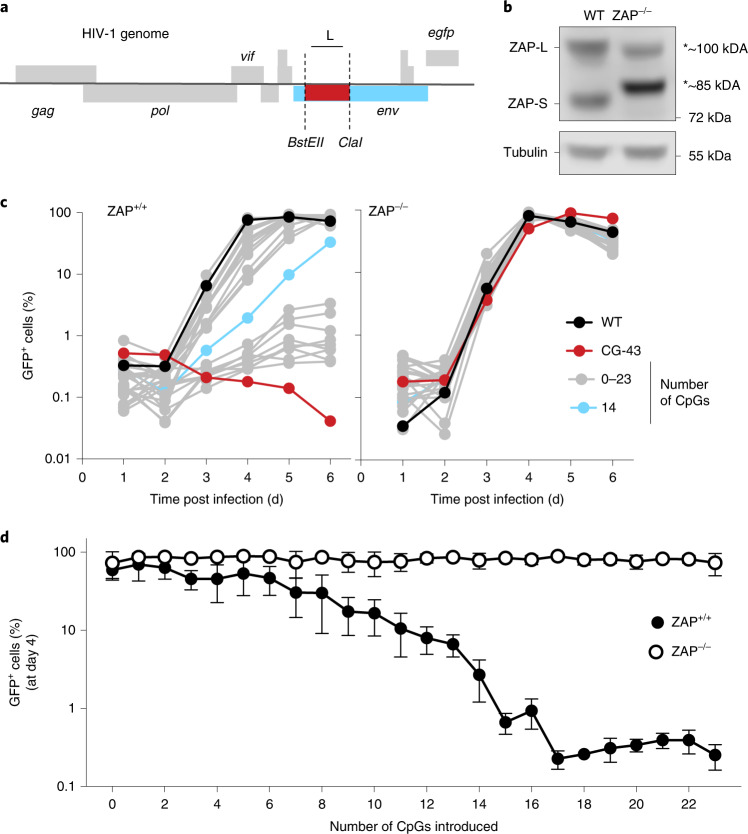
CpG dinucleotide numbers and HIV-1 replication. **a**, Schematic representation of HIV-1 genome containing an EGFP reporter in place of the *Nef* ORF and introduced restriction sites *BstEII* and *ClaI* in the 5′ portion of the *env* gene. **b**, ZAP proteins detected by western blotting in unmanipulated and functionally ZAP-deficient human MT4 T cells. The CRISPR lesion results in the expression of truncated non-functional ZAP proteins indicated by asterisks. **c**, Replication of a collection of HIV-1 mutants containing between 0 and 23 CpG dinucleotides, as well as WT and CG-43 in ZAP-expressing or ZAP-deficient cells. At each day after initial infection, a small sample of cells was collected and the percentage of GFP-positive cells was measured by flow cytometry. **d**, Percentage of infected cells at day 4 post initial infection across all mutant viruses. Mean ± s.d. from 3 independent experiments; two-way ANOVA for the presence of ZAP (column factor) *P* < 0.0001, number of CpG (row factor) *P* < 0.0001. Šídák’s multiple comparisons test was used to calculate adjusted *P* values between ZAP^+/+^ and ZAP^−/−^ groups, comparisons of virus mutants with more than 3 CpG display *P*_(adj)_ < 0.05. Source data

**Extended Data Fig. 1:**
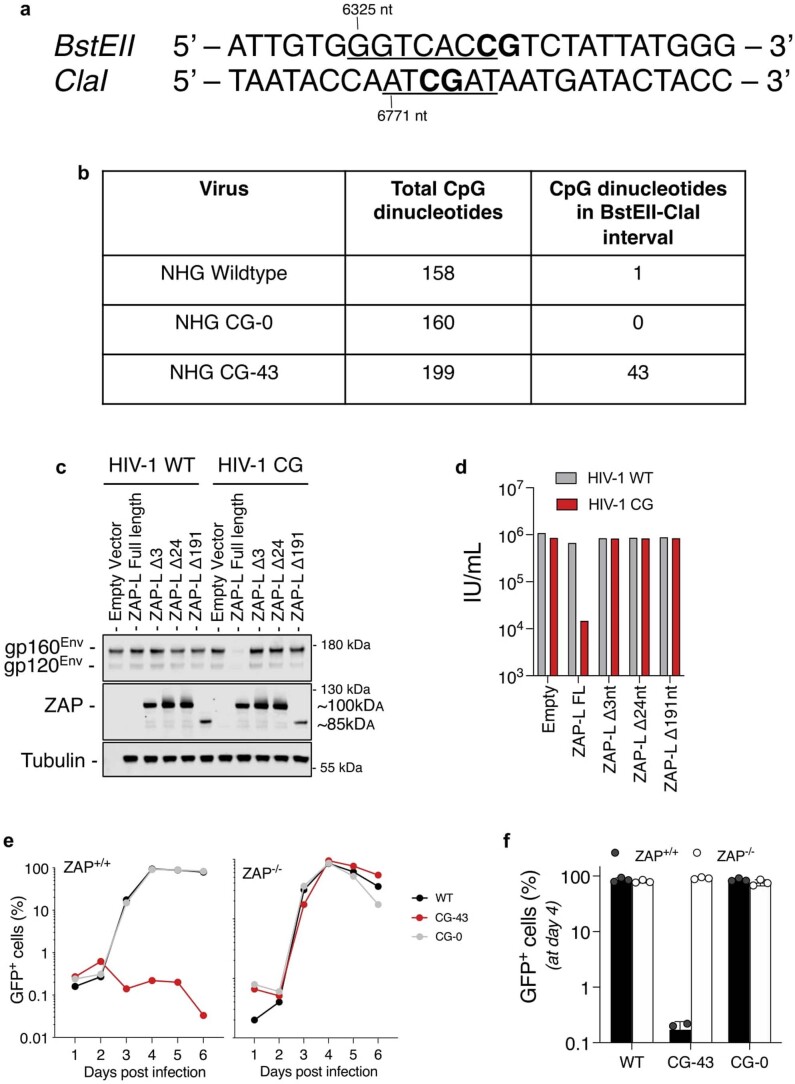
HIV-1 platform for analysis of CpG effects and ZAP-deficient MT4 cells. (**a**) Details of the insertion of *BstEII* and *ClaI* restriction sites at nucleotide positions 6325 and 6771, respectively, in the HIV-1 genome. (**b**) Summary of the number of CpG dinucleotides present in the HIV-1 reporter virus genome and in the *BstEII-ClaI* interval in *env*. (**c**) Western blot analysis of 293T cells cotransfected with HIV-1 (WT) or HIV-1 (CG-43) and plasmids expressing WT ZAP or the ZAP deletion mutants (Δ3, Δ24 and Δ191) recovered from the CRISPR/Cas9 edited MT4 cells. Blots are representative of 1 experiment. (**d**) Infectious virus yield from 293T cells cotransfected with HIV-1 (WT) or HIV-1 (CG-43) and plasmids expressing WT ZAP or the ZAP deletion mutants (Δ3, Δ24 and Δ191) recovered from the CRISPR/Cas9 edited MT4 cells. (**e**) Viral replication in ZAP-expressing and ZAP-knockout MT4 cells infected with wildtype (WT) or CG-43 mutant or a mutant containing the *BstEII* and *ClaI* restriction sites with 0 CpGs in the intervening region (CG-0). Each day, the percentage of infected cells was measured by flow cytometry. (**f**) Summary of the percentage of GFP-positive cells at day 4 post initial infection. Mean and SD from 3 independent experiments is plotted. Source data

HIV-1 is naturally CpG-poor and largely ZAP-resistant, while mutant derivatives with elevated CpG content are ZAP-sensitive^4^. We first introduced two unique restriction sites (*BstEII*-*ClaI)*, each of which contains a single CpG dinucleotide, into the HIV-1 *env* gene (Fig. 1a and Extended Data Fig. 1a). We synonymously recoded the intervening sequence, which lacks any known proximal *cis*-acting RNA regulatory elements, to contain zero (CG-0) or 43 (CG-43) CpG dinucleotides (Extended Data Fig. 1b). The CG-0 virus replicated indistinguishably from wild-type (WT) HIV-1 in both unmanipulated and in a CRISPR/Cas9-edited human MT4 T-cell line that expressed only exon 1-edited non-functional ZAP proteins. Conversely, the CG-43 virus replicated like WT virus in ZAP-deficient MT4 cells but was defective in ZAP-expressing cells (Fig. 1b and Extended Data Fig. 1c–f). Next, we generated a collection of HIV-1 mutants that differed from each other by a single CpG dinucleotide, with 1 to 23 CpG dinucleotides positioned as close to the *BstEII* as allowed by synonymous substitution in the *BstEII*-*ClaI* bounded region (CG-1 to CG-23). While all mutants replicated indistinguishably from WT HIV-1 in ZAP-deficient cells, virus replication was progressively diminished in ZAP-expressing cells as the number of CpG dinucleotides was increased (Fig. 1c,d). Overall, CG-1 to CG-13 replicated well, CG-15 to CG-23 replicated poorly and CG-14 had an intermediate phenotype (Fig. 1c). The percentage of infected cells at 4 d post infection showed an obvious correlation between the number of introduced CpG dinucleotides and the extent of replication (Fig. 1d). Thus, individual CpG dinucleotides had an incremental impact, and approximately 15 CpG dinucleotides were required to profoundly inhibit HIV-1 replication.

### Spacing and base composition between CpG dinucleotides and ZAP activity

**Fig. 2:**
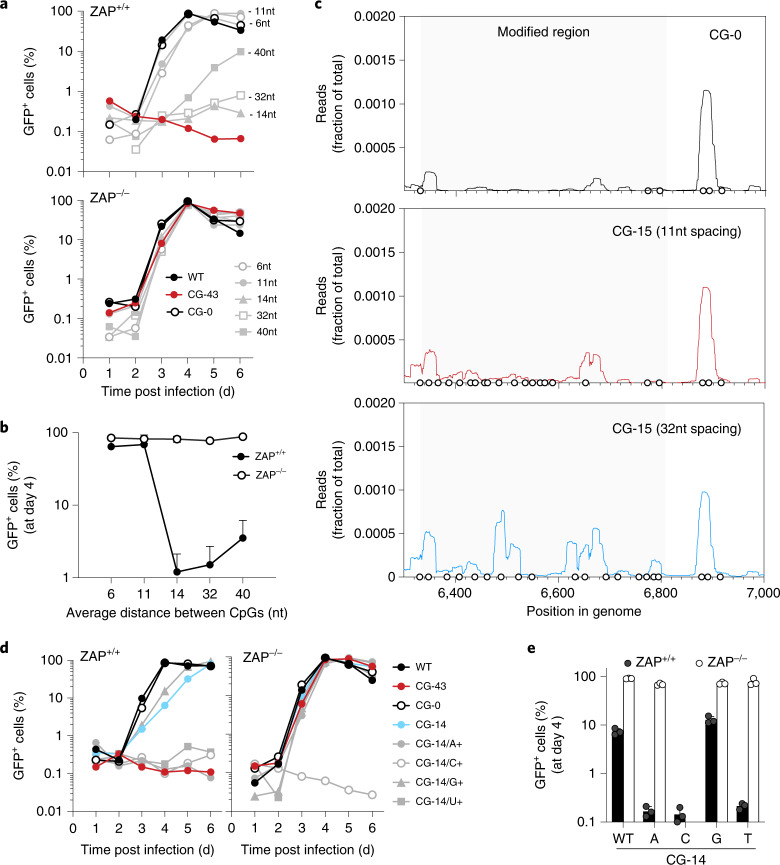
Spacing and composition between CpG dinucleotides affect HIV-1 replication. **a**, Viral replication in cells infected with mutants of HIV-1 GFP reporter viruses containing 15 additional CpG dinucleotides with a mean of 6, 11, 14, 32 or 40 nucleotides between each CpG dinucleotide. Each day, the percentage of infected cells was measured by flow cytometry. **b**, Summary of the percentage of GFP-positive cells at day 4 post initial infection. Mean ± s.d. from 3 independent experiments; two-way ANOVA for the presence of ZAP (column factor) *P* < 0.0001, spacing (row factor) *P* = 0.002. Šídák’s multiple comparisons test was used to calculate adjusted *P* values between ZAP^+/+^ and ZAP^−/−^ groups, comparisons of virus mutants with spacing between CpGs ≥14 nt display *P*_(adj)_ < 0.0001. **c**, CLIP-seq analysis of ZAP binding to HIV-1 RNA: ZAP-deficient and TRIM25-deficient 293T cells were transfected with a plasmid encoding ZAP-L as well as HIV-1 proviral plasmids with 0 CpG dinucleotides (black line), or 15 CpG dinucleotides with average spacing of 11 nucleotides (red line) or 32 nucleotides (blue line) in the *BstEII-ClaI* interval. CLIP reads that mapped to the *BstEII-ClaI* interval are plotted as the normalized fraction of total reads. Circles indicate the positions of CpG dinucleotides. **d**,**e**, Replication of CG-14/A+/U+/C+/G+ mutant viruses in ZAP-expressing and ZAP-deficient MT4 cells. The percentage of GFP-positive cells was measured daily (**d**) and the mean ± s.d. of 3 independent experiments were measured at day 4 post infection (**e**). Source data

We generated a second collection of HIV-1 mutants that each contained 15 CpG dinucleotides but differed in the spacing between each CpG dinucleotide. In ZAP-expressing cells, viruses that contained 15 CpG dinucleotides separated by a mean of 6 or 11 nucleotides replicated with near-WT kinetics. Conversely, viruses with CpG dinucleotides separated by 14 or 32 nucleotides were defective (Fig. 2a,b), while the effect of the 15 CpG dinucleotides was diminished if the spacing between them was further increased to a mean of 40 nucleotides.

To examine ZAP binding to these mutant viral sequences, we performed crosslinking immunoprecipitation assays coupled with RNA sequencing (CLIP-seq) using viruses with 15 CpG dinucleotides, separated by a mean of 11 or 32 nucleotides. We measured the frequency of CLIP-seq reads mapping to each nucleotide position in the *BstEII*-*ClaI* interval of the HIV-1 genome. While viral RNA containing no CpG dinucleotides in the *BstEII*-*ClaI* interval showed little ZAP binding, there was abundant ZAP binding when the CpG dinucleotides were positioned at a mean of 32 nucleotides apart (Fig. 2c). However, ZAP binding to the modified sequence was minimal when CpG dinucleotides were positioned at a mean of 11 nucleotides apart (Fig. 2c). Thus, these data suggest that adequate spacing between CpG dinucleotides is important for ZAP recognition.

**Extended Data Fig. 2:**
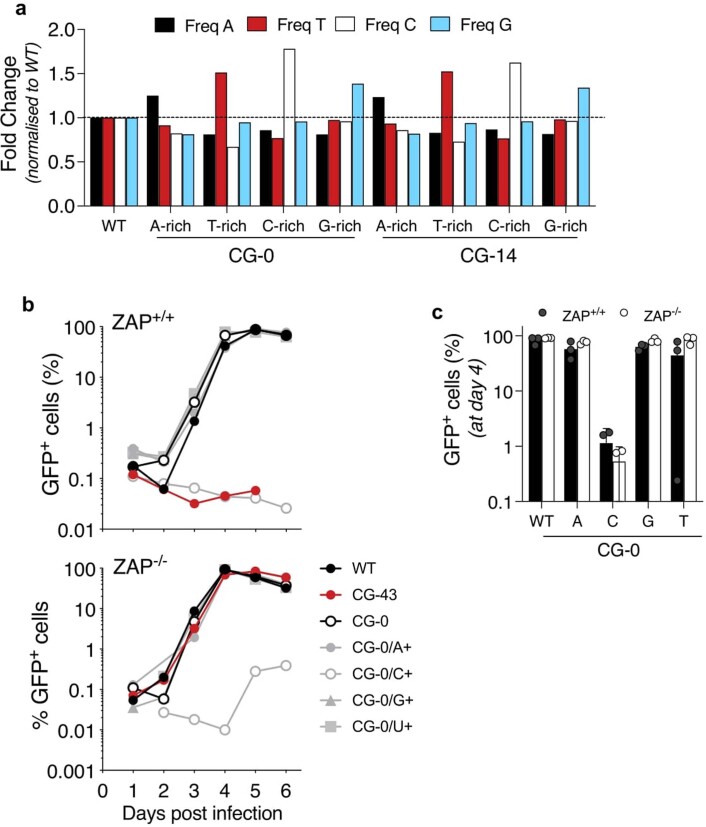
Mononucleotide composition of HIV-1 mutants. (**a**) Summary of mononucleotide composition changes relative to the wildtype HIV-1 in the *BstEII-ClaI* interval for CG-0/A+/U+/C+/G+ and CG-14/A+/U+/C+/G+ mutants. (**b**, **c**) Replication of CG-0/A+/U+/C+/G+ mutant viruses in ZAP-positive and ZAP-negative MT4 cells. The percentage of GFP positive cells was measured daily (**b**) and the mean and SD of 3 independent experiments measured at day 4 post infection (**c**). Source data

Based on previous CLIP-seq experiments^4,13^ and ZAP–RNA crystal structures^14,15^, ZAP specificity is determined solely by the target CpG dinucleotide and not flanking nucleotides. However, whether the overall sequence context in which CpG dinucleotides are present contributes to ZAP antiviral activity is unknown. We generated HIV-1 mutants that contained either 0 or 14 CpG dinucleotides (CG-0 and CG-14) and synonymously mutated the surrounding sequence in the *BstEII*-*ClaI* interval to contain the maximum possible number of adenine (A+), cytidine (C+), guanine (G+) or uridine (U+) nucleotides (Extended Data Fig. 2a). The CG-0 viruses with elevated A, U or G content (CG-0/A+, CG-0/U+ and CG-0/G+) replicated with close to wildtype kinetics, while the cytidine-enriched virus (CG-0/C+) showed severe replication defects, independent of the presence of ZAP (Extended Data Fig. 2b,c). The CG-14/A+, CG-14/U+ and CG-14/G+ viruses replicated similarly to wild type in ZAP-deficient cells while the CG-14/C+ virus exhibited a ZAP-independent defect similar to the CG-0/C+ virus (Fig. 2d). Notably, while elevating G content (CG-14/G+) had little impact on virus replication, the CG-14/A+ and CG-14/U+ viruses were severely attenuated, specifically in ZAP-expressing cells (Fig. 2d,e). Thus, increasing A or U content apparently increased the ability of CpG dinucleotides to impart ZAP sensitivity.

**Extended Data Fig. 3:**
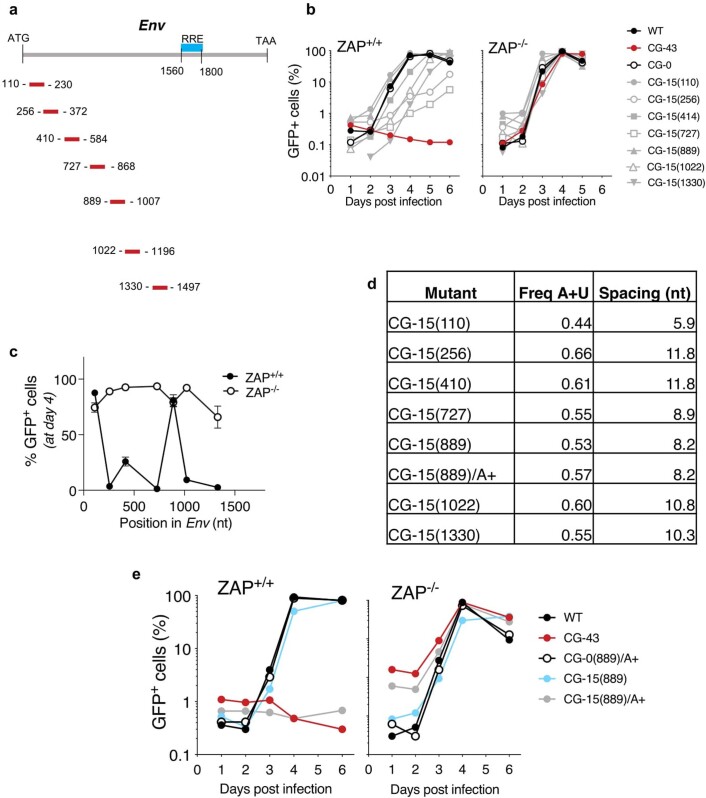
Effect of genome position in ZAP antiviral activity. (**a**) Diagram of the location of the mutated sequence in the *env* gene. Rev response element (RRE) is located between nucleotides 1560 and 1800. Position coordinates for each mutant is indicated by the location of the first and last CpG dinucleotides. (**b**) Replication of a collection of HIV-1 mutants (CG-15(110) through CG-15(1330)) each containing 15 CpG dinucleotides at different positions in the *env* gene as indicated in (a). Numbers in parentheses indicate the nucleotide position within *env* of the first introduced CpG. Percentage of infected cells was monitored by flow cytometry. (**c**) Summary of percentage of infected cells 4 days post infection, mean and s.d. of three experiments is plotted. (**d**) Summary of overall A and U content in the mutated regions of the mutants depicted in (a) (plus 10 nucleotides 5′ and 3′ of the first and last CpG, respectively) and mean number of nucleotides between each CpG. nt, nucleotide. (**e**) Replication of CG-15(889) recoded to contain high levels of adenine (CG-15(889)/A+). Cells were infected and infection was monitored as in (**a**). Source data

We next generated 7 HIV-1 mutants, each containing 15 additional CpG dinucleotides, with the CpG-enriched sequences positioned at different locations across the *env* gene (Extended Data Fig. 3a). All these viruses replicated similarly to WT HIV-1 in ZAP-deficient cells, and 5/7 exhibited ZAP-dependent attenuation. The exceptions were two viruses with CpG-enriched segments located 3′ to *env* nucleotide positions 110 or 889 (CG-15(110) and CG-15(889), Extended Data Fig. 3b,c). Notably, the A and U frequencies in these two regions were reduced compared with other HIV-1 genome regions, and the mean spacing between CpG dinucleotides was the lowest among the mutants (Extended Data Fig. 3d). Strikingly, increasing the adenine frequency of CG-15(889) in the CpG-enriched interval to generate CG-15(889)/A+ increased ZAP-dependent attenuation such that CG-15(889)/A+ was specifically defective in ZAP-expressing cells (Extended Data Fig. 3d,e). We conclude that apparent position-dependent effects on ZAP sensitivity are probably mediated by surrounding nucleotide composition.

### Selective pressure by ZAP can deplete CpG dinucleotides from viral genomes

The paucity of CpG dinucleotides in mammalian virus genomes may have been driven by ZAP selection^13,19^. The potential utility of reversing this property to generate ZAP-sensitive, live-attenuated vaccines depends on the stability of attenuating mutations. While codon-pair deoptimization has been reported to be stable during in vitro passage^20,21^, the stability of introduced CpG dinucleotides during viral passage under selective pressure by ZAP has not been assessed.

**Fig. 3:**
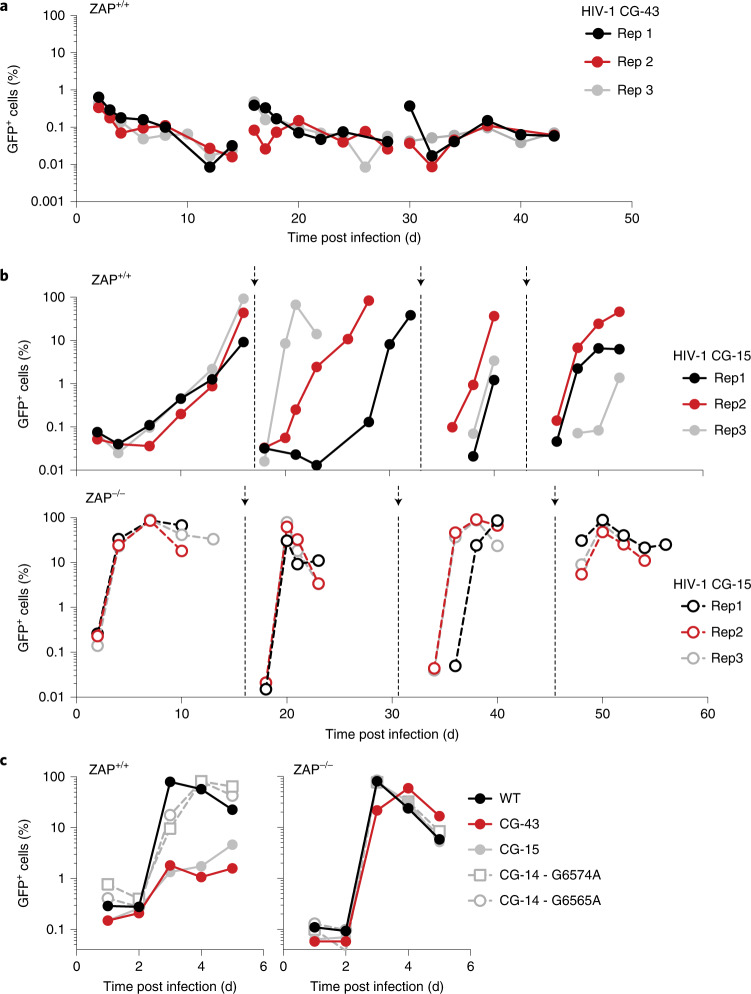
ZAP can impose selective pressure to deplete CpG dinucleotides. **a**, Replication, monitored by flow cytometry, of the CG-43 HIV-1 mutant in unmanipulated MT4 cells in 3 replicate cultures (Rep1–3). **b**, Replication of the CG-15 HIV-1 mutant in MT4 cells in 3 replicate cultures. Supernatants were collected, filtered and used to infect a new culture of MT4 cells when the percentage of infected cells exceeded 80%, as indicated by arrows. **c**, Replication of the CG-15 HIV-1 mutant with re-introduced mutations (G6574A or G6565A) in ZAP^+/+^ and ZAP^−/−^ cells. Source data

**Extended Data Fig. 4:**
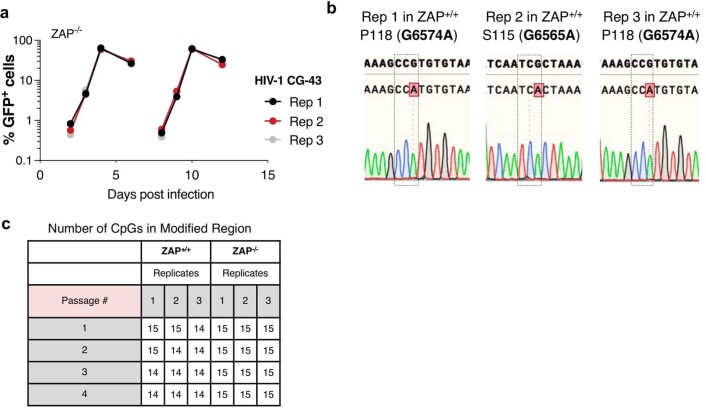
Replication and monitoring of CpG reversion during passage of CpG enriched HIV-1. (**a**) Replication of the CG-43 HIV-1 mutant in ZAP-deficient MT4 cells in three replicate cultures (Rep1-3). Percentage of infected cells was monitored by flow cytometry. (**b**, **c**) Sequence analysis of CG-15 HIV-1 mutant during replication. Unique point mutations in each replicate (**b**) and the total number of CpGs in each replicate at each passage (**c**) are indicated. Source data

We performed long-term serial passage experiments with HIV-1 mutants containing 15 or 43 CpG dinucleotides (CG-15 and CG-43). Replication of CG-43 was severely inhibited in ZAP-expressing cells over the entire course of the experiment (43 d). Indeed, CG-43 never infected more than 1% of the cell population and no cytopathic effects typical of HIV-1 replication were observed (Fig. 3a and Extended Data Fig. 4a). Conversely, the CG-15 virus initially replicated poorly in ZAP-expressing cells but was able to rapidly infect most of the cell population at later passages (Fig. 3b). Sequence analyses revealed a synonymous G-to-A transition at the wobble position of either a proline or a serine codon that caused the loss of a single CpG dinucleotide in each of the CG-15 experimental replicates, associated with increased replication (Extended Data Fig. 4b,c). Re-introducing these acquired point mutations into the parental CG-15 genome showed that the loss of a single CpG dinucleotide caused fitness recovery of CG-15 in ZAP-expressing cells (Fig. 3c). We conclude that attenuation of HIV-1 through introduction of a large number of CpG dinucleotides presents difficult-to-surmount genetic and stable attenuation barriers. Conversely, the presence of a number of CpG dinucleotides close to a threshold level enables emergence of ZAP-insensitive variants when selection pressure is applied.

### Engineering enterovirus A71 to confer ZAP sensitivity

**Fig. 4:**
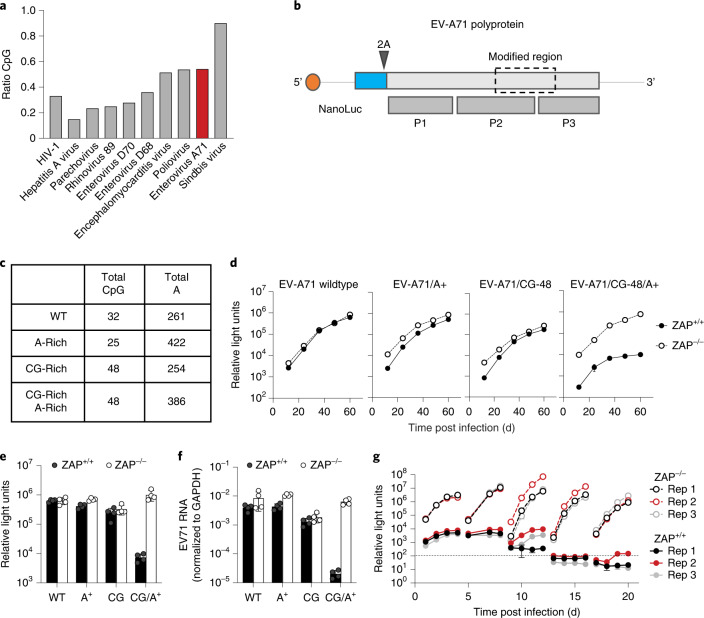
Genomic recoding sensitizes EV-A71 to ZAP. **a**, CpG content of example virus genomes; observed/expected ratios (based on mononucleotide composition) for HIV-1, Sindbis virus and several picornaviruses. **b**, Schematic diagram of reporter EV-A71 containing the NanoLuc luciferase gene (blue) and a 2A cleavage site. P1, P2 and P3 indicate the primary processed proteins derived from the EV-A71 polyprotein. The recoded region (dashed line box) is approximately 1 kb and encodes a portion of the polyprotein located in P2 and P3. **c**, Summary of the recoding modifications introduced in the EV-A71 genome. **d**–**f**, Replication of reporter EV-A71 mutants in ZAP^+/+^ or ZAP^−/−^ HeLa cells. NanoLuc luciferase activity was measured every 12 h (**d**). Summary of NanoLuc luciferase levels at 60 h post infection, mean ± s.d. of 3 independent experiments (**e**). Viral RNA levels measured at 60 h post infection, mean ± s.d. of 3 independent experiments (**f**). **g**, Replication of EV-A71(CG-48)/A+ in ZAP^+/+^ or ZAP^−/−^ HeLa cells over 4 passages. At 4 d post initial infection, supernatants were collected, filtered through a 0.22 µm filter and used to infect a fresh culture of cells. Source data

Members of the *Picornaviridae* are important human pathogens that cause morbidity^22^ and most lack efficacious vaccines. One such picornavirus is enterovirus A71 (EV-A71) that causes hand, foot and mouth disease in young children, with occasional severe complications including acute flaccid paralysis, brainstem encephalitis and meningitis^23^. EV-A71 has a low frequency of CpG dinucleotides (Fig. 4a) and is therefore a good candidate for genetic recoding to confer ZAP sensitivity and generate a potential live-attenuated vaccine.

The EV-A71 genome encodes a single polyprotein (Fig. 4b) that is cleaved to generate structural (VP1, VP2, VP3 and VP4) and non-structural proteins (2A, 2B, 2C, 3A, 3C, VPg and the RNA-dependent RNA polymerase). To monitor EV-A71 replication, we generated a reporter virus encoding NanoLuc luciferase followed by a 2A cleavage site at the N terminus of the viral polyprotein, as previously described^24^. For recoding, we applied the CpG number, spacing and intervening mononucleotide content criteria determined above using HIV-1. We recoded a ~1 kb target region that spanned 2C, 3A, VPg and 3C coding sequences and was selected arbitrarily, other than the fact that it lacks any known proximal *cis*-acting RNA regulatory elements (Fig. 4b). In WT EV-A71, this region contains 32 CpG dinucleotides and 261 adenines (Fig. 4c). We changed the number and distribution of CpG dinucleotides, as well as the adenine content, generating three mutants: (1) EV-A71/A+ that contained a high frequency of A nucleotides without increasing the number of CpG dinucleotides; (2) EV-A71/CG-48 with 16 additional CpG dinucleotides that combined with the existing 32 CpG dinucleotides, generating a segment with 48 CpG dinucleotides at a mean of 19 nucleotides apart but retaining WT mononucleotide composition; and (3) EV-A71/CG-48/A+ that had the 16 additional CpG dinucleotides positioned as above but in the A-rich sequence context (Fig. 4c). All viruses replicated well in ZAP-deficient cells (Fig. 4d–f). While no replication defects were observed in the WT EV-71, EV-A71/A+ or EV-A71/CG-48 mutants, the EV-A71/CG-48/A+ mutant was specifically attenuated in ZAP-expressing cells (Fig. 4d–f).

**Extended Data Fig. 5:**
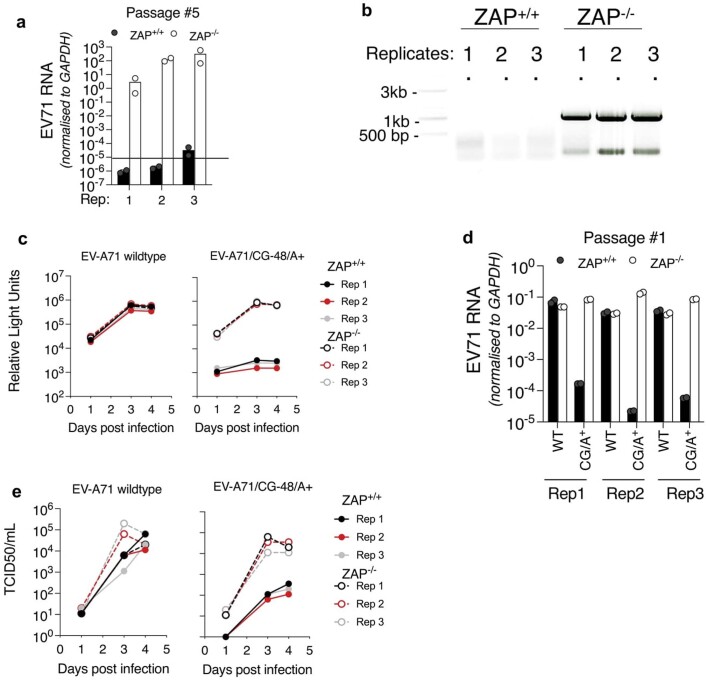
Replication and monitoring of CpG reversion during passage of CpG enriched EV-A71. (**a**) EV-A71 RNA levels measured at the end of passage #5, 60 h post-infection in three replicate cultures (mean and s.d. of 3 replicates is plotted). (**b**) PCR amplification of the modified region of EV-A71/CG-48/A+ in three replicates at the end of a long-term passage in ZAP^+/+^ and ZAP^−/−^ cells (amplified region is approximately 1 kb). (**c**–**e**) Replication of EV-A71 wildtype and mutant reporter viruses in ZAP^+/+^ and ZAP^−/−^ HeLa cells. NanoLuc luciferase activity was measured every 12 h (**c**), or quantification of viral RNA at day 4 (**d**), mean and s.d. plotted; data from 3 replicates. Alternatively replication of EV-A71 wildtype and mutant viruses monitored by TCID50 measurement at each timepoint (**e**); 3 replicates shown. Source data

When the EV-A71/CG-48/A+ mutant was repeatedly passaged in ZAP-expressing cells, luciferase activity progressively decreased with each passage and was ultimately below the detection limit (Fig. 4g). Conversely, no replication defect was evident in ZAP-deficient cells, and sequence analyses revealed no reversion mutations acquired during passage. While viral RNA was abundant in all EV-A71/CG-48/A+ replicates in ZAP-deficient cells, RNA levels were below the detection limit in ZAP^+/+^ cells (Extended Data Fig. 5a,b). Moreover, when we followed virus replication for 4 days using NanoLuc assays, determination of infectious virus yield (TCID_50_) and viral RNA quantification, all three measurements of EV-A71/CG-48/A+ mutant replication were dramatically reduced in ZAP^+/+^ cells compared with ZAP-deficient cells (Extended Data Fig. 5c–e). Thus, the observed replication deficits reflected bona fide effects of ZAP and not reporter gene instability. Moreover, the CpG and A enrichment was stable and EV-A71/CG-48/A+ could not escape ZAP under these conditions. Thus, the principles governing ZAP sensitivity identified using HIV-1 could be applied to an unrelated RNA virus, leading to stable, ZAP-dependent attenuation.

### ZAP-dependent attenuation of recoded EV-A71 in mice

**Fig. 5:**
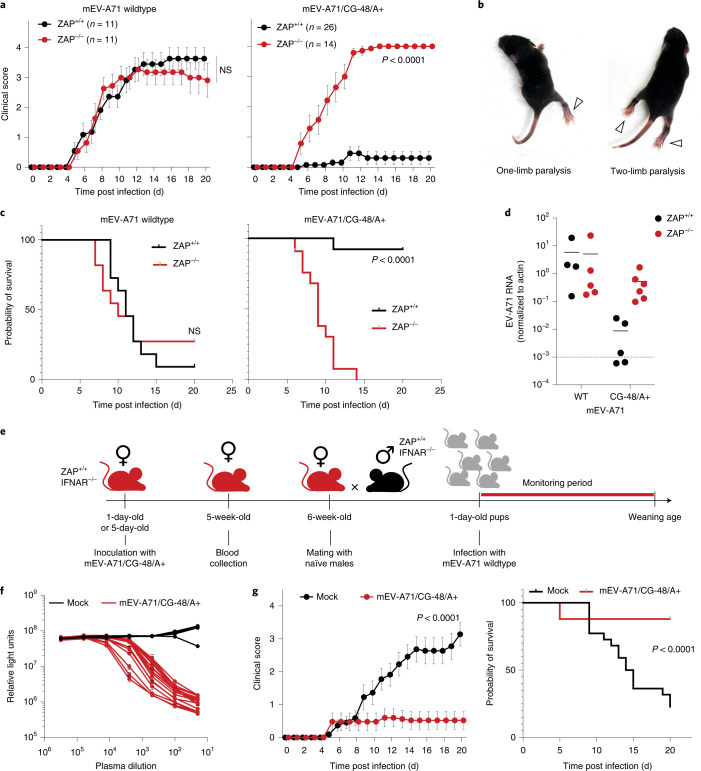
Recoded EV-A71 has ZAP-dependent attenuation and elicits protective antibodies in mice. **a**, Clinical score following infection of 1-day-old ZAP^+/+^ and ZAP^−/−^ mice with mEV-A71 WT or mEV-A71/CG-48/A+ (mean ± s.d.; *n* = 11–26 mice per group; *P* values calculated using two-way ANOVA; NS, non-significant). **b**, Examples of characteristic pathology developed in infected mice following mEV-A71 infection, including one-limb and two-limb paralysis. **c**, Probability of ZAP^+/+^ and ZAP^−/−^ mouse survival (%) following infection with mEV-A71 WT or mEV-A71/CG-48/A+ (*n* = 11–36 mice per group; *P* value calculated using Mantel-Cox test). **d**, One-day-old mice (*n* = 4–6 per group) were infected with indicated virus; 6-days-post-infection mice were sacrificed and muscles from both posterior limbs were collected, homogenized and total RNA was extracted. EV-A71-specific RNA was quantified by qPCR. Dashed line indicates limit of qPCR detection. **e**, Schematic representation of experimental design. ZAP^+/+^Ifnar^−/−^ female mice previously inoculated with mEV-A71/CG-48/A+ (or mock-infected females) were mated with *ZAP*^+/+^*Ifnar*^−/−^ male mice. Resulting offspring was challenged at 1 d of age with mEV-A71 WT. **f**, Neutralizing activity in plasma from mice after mEV-A71/CG-48/A+ infection (infection at 1 d of age), blood collection at 5 weeks, or mock-infected mice evaluated using EV-A71 NanoLuc luciferase reporter virus. 293T cells were incubated with the antibody:virus mixture for 48 h and luciferase activity was measured. **g**, Clinical score and survival probability following mEV-A71 (WT) infection of the offspring of ZAP^+/+^/IFNAR^−/−^ females previously inoculated with mEV-A71/CG-48/A+ (*n* = 25 pups from 4 different females) or previously mock-infected (*n* = 22 pups from 4 different females) at 1 d of age. Clinical score and survival were assessed daily until weaning. Statistical significance was inferred by two-way ANOVA and Mantel-Cox tests. Source data

**Extended Data Fig. 6:**
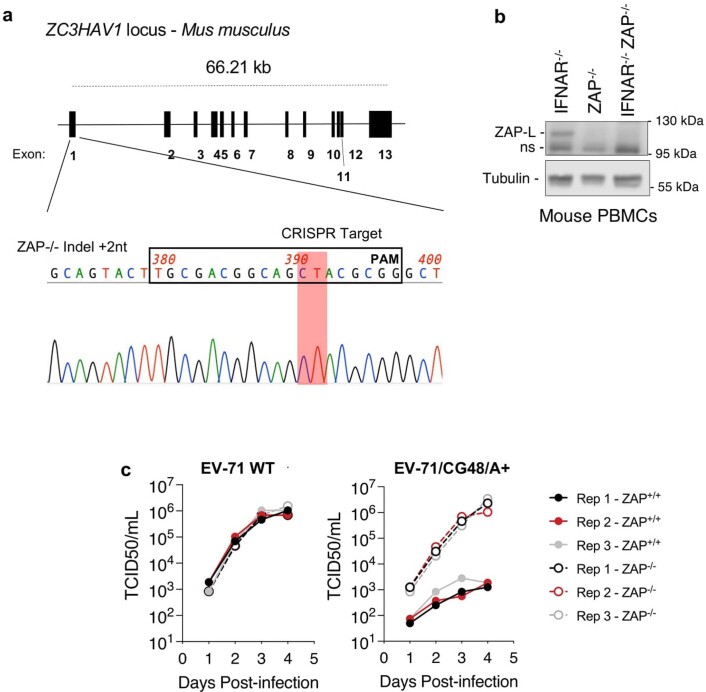
Generation of a ZAP knockout mouse line and ZAP-dependent attenuation of EV-A71 lacking the NanoLuc reporter. (**a**) Schematic diagram of the ZC3HAV1 locus in the *Mus musculus* genome. Sequence chromatogram of CRISPR target site PCR product amplified from homozygous ZAP^−/−^ mice. A frameshift mutation in the essential N-terminal RNA binding domain via the insertion of two nucleotides is indicated. (**b**) Western blot analysis of mouse peripheral blood mononuclear cells (PBMCs) isolated from ZAP^+/+^IFNAR^−/−^ and ZAP^−/−^IFNAR^−/−^ mouse lines. (**c**) Replication of EV71 WT and EV71/CG48/A+ in ZAP^+/+^ and ZAP^−/−^ HeLa cells, monitored by TCID50 measurement at each timepoint; 3 replicates are shown. Source data

**Extended Data Fig. 7:**
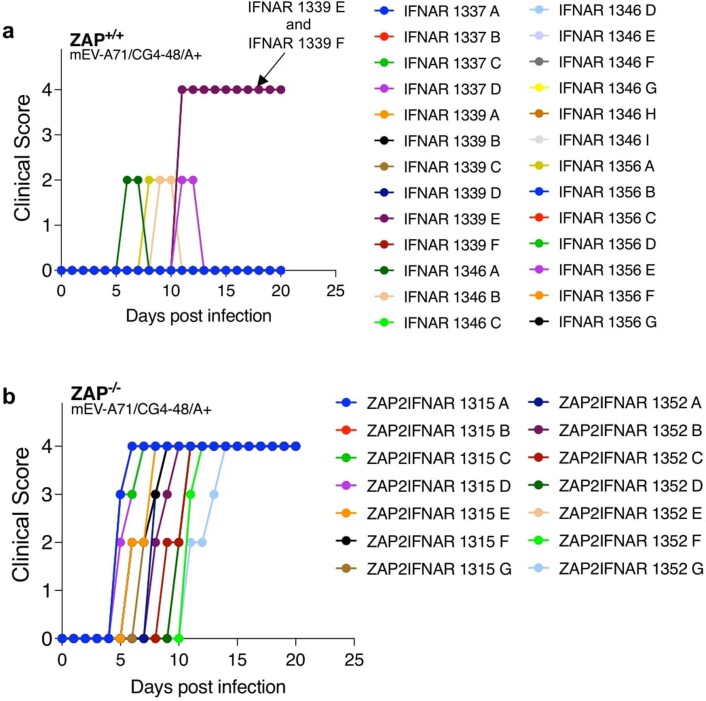
Clinical score in individual mEV71/CG48/A+infected ZAP^+/+^ and in ZAP^−/−^ mice. (**a**, **b**) Clinical scores of individual mice following infection of one-day old ZAP^+/+^ (**a**) and ZAP^−/−^ (**b**) mice with mEV-A71/CG-48/A+. Source data

To determine whether ZAP could inhibit EV-A71/CG-48/A+ replication in vivo, we generated a ZAP^−/−^ C57BL/6 mouse line using CRISPR guide RNAs targeting *ZC3HAV1* exon 1. A germline-transmissible edited ZAP allele contained a 2 nt insertion, introducing a frameshift mutation that abrogated ZAP expression (Extended Data Fig. 6a,b). We introduced substitutions in EV-A71, these substitutions being required for symptomatic infection of mice^25^, thus generating ‘mouse-adapted’ viruses, hereafter referred to as mEV-A71 and mEV-A71/CG-48/A+. These viruses lacked the NanoLuc reporter, but EV-A71/CG-48/A+ exhibited the same ZAP-dependent replication deficits as the aforementioned NanoLuc encoding EV-71 reporter constructs (Extended Data Fig. 6c). Because we observed more consistent mEV-A71 pathogenesis in IFNAR^−/−^ neonatal mice, we crossed the ZAP^−/−^ C57BL/6 mouse line to an IFNAR^−/−^ C57BL/6 mouse line. We then infected ZAP^+/+^/IFNAR^−/−^ or ZAP^−/−^/IFNAR^−/−^ neonates with mEV-A71 or mEV-A71/CG-48/A+ and scored disease progression according to a previously described 0–4 scale; from asymptomatic (0) to dead or moribund (4)^26^ (Fig. 5a). Limb paralysis events (Fig. 5b) characteristic of EV-A71 infection in similar mouse models^26,27^ were observed upon mEV-A71 infection of either ZAP^+/+^ or ZAP^−/−^ mice, with disease progression that was ultimately fatal in most cases (Fig. 5a–c). Conversely, while ZAP^−/−^ mice succumbed to the mEV-A71/CG-48/A+ virus, nearly all ZAP^+/+^ mice survived infection and presented with low clinical scores (Fig. 5a–c). Specifically, 20/26 mEV-A71/CG-48/A+ infected ZAP^+/+^ mice showed no symptoms, while 4 mice exhibited enterovirus-specific symptoms (that is, limb paralysis) that quickly resolved (Extended Data Fig. 7a). Two ZAP^+/+^ mice from a single litter died suddenly without limb paralysis, suggesting that their death was not due to enteroviral disease. In contrast, all mEV-A71/CG-48/A+ infected ZAP^−/−^ mice developed enterovirus-specific symptoms before death (Extended Data Fig. 7b). Viral RNA levels in muscle were equivalent in mEV-A71-infected ZAP^+/+^ and ZAP^−/−^ mice but 60-fold lower in mEV-A71/CG-48/A+-infected ZAP^+/+^ mice compared with ZAP^−/−^ mice (Fig. 5d). Sequences of PCR amplicons encompassing the engineered regions of the mEV-A71/CG-48/A+ genome from all mice in which viral RNA was detectable (Fig. 5d) revealed no mutations. Together, these data indicate that mEV-A71/CG-48/A+ was strongly and stably attenuated in vivo, and its attenuation was strictly dependent on ZAP.

### Recoded EV-A71 elicits protective immunity in mice

**Extended Data Fig. 8:**
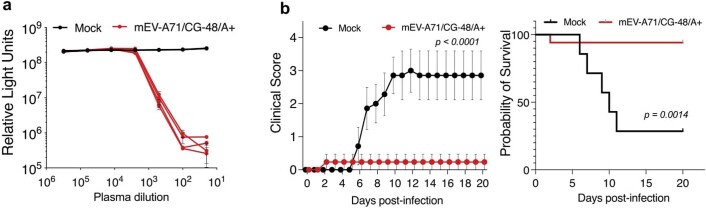
mEV-A71/CG-48/A+ elicits protective antibodies in ZAP^+/+^mice. (**a**) Neutralizing activity in plasma from mice after mEV-A71/CG-48/A+ infection (n = 4, at day 5 of age, blood collection at 6-weeks) or mock-infected mice (n = 3) evaluated using EV-A71 NanoLuc luciferase reporter virus. 293T cells were infected with the antibody:virus mixture, incubated for 48 h and luciferase activity was measured. Mean and SD plotted. (**b**) Clinical score and survival probability following mEV-A71 (wildtype) infection of the offspring of ZAP^+/+^IFNAR^−/−^ females previously inoculated with mEV-A71/CG-48/A+ (n = 17 pups from 3 different females) or mock-infected (n = 7 pups from 1 female) at day 5 of age. Clinical score and survival were assessed daily until weaning age. Mean and SEM plotted. Source data

We collected plasma from ZAP^+/+^ mice 5 weeks after previous inoculation with mEV-A71/CG-48/A+ at 1 d or 5 d of age (Fig. 5e). While plasma from mock-inoculated mice did not neutralize EV-A71, plasma from mice inoculated with mEV-A71/CG-48/A+ neutralized EV-A71 infection, with 50% neutralizing titres (NT_50_) ranging from 895 to 10,602 in mice infected at 1 d of age (median NT_50_ = 2,957) and titres ranging from 627 to 1,514 in mice infected at 5 d of age (median NT_50_ = 1,162) (Fig. 5f and Extended Data Fig. 8a). Next, we aimed to determine whether these neutralizing antibodies were protective in vivo. Since productive infection of mEV-A71 is age-sensitive in mice^28^, we performed passive protection experiments in which the neonatal offspring of females that were previously mock-inoculated or inoculated with mEV-A71/CG-48/A+ were challenged with WT mEV-A71 (Fig. 5e). In this type of experiment, suckling pups acquire antibodies via maternal milk^26^. Pups from females previously inoculated with mEV-A71/CG-48/A+ at 1 d or 5 d of age (Fig. 5e) showed reduced disease (median clinical score at day 20 of 0.52 and 0.24 in 1-day-old and 5-day-old infected mice, respectively) and increased survival compared with pups from mock-treated females (median clinical score at day 20 of 3.14 and 2.86 in 1-day-old and 5-day-old infected mice, respectively) (Fig. 5g and Extended Data Fig. 8b). We conclude that ZAP-attenuated mEV-A71/CG-48/A+ replication in mice elicits antibodies that are passively transferred and protective against mEV-A71 disease in the offspring of inoculated females.

## Discussion

The delineation of sequence features that affect sensitivity to ZAP in HIV-1 (numbers of CpG, spacing and context) enabled us to develop design rules that we applied to engineer a picornavirus mutant that is strongly attenuated in a strictly conditional manner. The close spacing between each CpG dinucleotide affecting ZAP sensitivity and ZAP binding in CLIP-seq suggests that ZAP molecules binding to adjacent CpG dinucleotides may compete with each other, consistent with modelling studies indicating that ZAP binds RNA sequences of ~13 nucleotides^29^. Closely spaced CpG dinucleotides may also promote RNA secondary structure that might inhibit ZAP access^30^. Conversely, wide CpG spacing conferred reduced sensitivity. Interactions between ZAP and TRIM25^13^ and between ZAP and KHNYN^31^, two known co-factors of ZAP, appear to be mediated by protein-protein contacts; thus, it is possible that ZAP molecules and co-factors bound to adjacent CpG dinucleotides may coalesce, with close spacing facilitating assembly of active ZAP:TRIM25:KHNYN complexes.

Studies of RNA-binding protein specificity typically focus on the recognition of particular RNA sequences. Although many RNA-binding proteins recognize specific sequence motifs, contextual features, such as flanking nucleotide composition, can be crucial for determining target specificity^32^. Increasing adenine or uridine content in viral genomes may increase sensitivity to RNAses, such as RNAse L that cleaves UpA and UpU dinucleotides in viral RNA, and we note that our A- or U-enriched viral genomes contain greater numbers of UpA dinucleotides^33,34^. Nevertheless, A/U-enriched viruses replicated identically to wildtype HIV-1 in ZAP-deficient cells, indicating that the defects imposed by A/U enrichment are ZAP specific. An obvious effect of A or U enrichment would be to reduce stable secondary RNA structure, perhaps increasing CpG dinucleotide accessibility to ZAP.

All approaches to viral attenuation must balance reduced pathogenesis versus reduced antigen levels that accompany impaired viral genome expression and replication. In principle, programed attenuation of viruses based on ZAP sensitivity might be adjustable through variation in CpG number and accessibility. Because RNA features conferring ZAP sensitivity in HIV-1 were readily transferrable to a very different virus (EV-A71), these approaches may be generally applicable, and it is possible that nearly any virus that exhibits CpG depletion could be a suitable target for this recoding approach. Importantly, no loss-of-function mutations in ZAP have been identified so far in humans, but further investigation would be required to evaluate whether existing genetic variation in ZAP might render some individuals more susceptible to CpG-enriched viruses and whether the stability of the introduced mutations that we observed for HIV-1 and EV-A71 in cell culture and in mice would be generalizable to CpG-enriched virus-vaccinated humans. Attenuation through engineered ZAP sensitivity might be ineffective for viruses with alternative mechanisms of ZAP evasion, such as targeting ZAP or co-factors for depletion, although no such viruses are currently known. Notably, strictly ZAP-dependent attenuation allows for the cultivation of high-titre, live-vaccine stocks in ZAP-deficient cells. Additional advantages may stem from the reported observation that RNA recognition by ZAP is immunostimulatory^18^. Indeed, infection of mice with CpG-enriched influenza A viruses elicited immune responses that were disproportionate to the level of virus replication^35^. ZAP-independent attenuation through unguided deoptimization may forego these benefits. We also note that the principles described herein may also prove useful in the engineering of nucleic acid-based gene delivery vectors. Indeed, CpG dinucleotides depletion from the DNA of gene delivery vectors was reported to improve performance^36,37^.

In summary, our results identify sequence features that are important for recognition of foreign RNA by ZAP and thereby enable highly specific rational attenuation of two different RNA viruses. Our findings establish design principles for engineering CpG-enriched viruses that are conditionally attenuated and can elicit a protective immune response in mice, thereby paving the way to the rational design of live-attenuated viruses with vaccine potential.

## Methods

### Cells and animals

Human embryonic kidney (HEK) 293T ZAP^−/−^ TRIM25^−/−^ cells^13^, HeLa LCV1 (non-targeting control) and ZAP^−/−^ cells^4^ were cultured in Dulbecco’s modified Eagle medium (DMEM) supplemented with fetal bovine serum (FBS) and gentamycin. A ZAP^−/−^ cell line based on RD cells was generated using CRISPR-Cas9 as previously described^4^ and a guide RNA targeting exon 1 of human ZAP (5′-GGCCGGGATCACCCGATCGGTGG-3′). MT4 LCV1 and MT4 B1 ZAP^−/−^ cell lines^4^ were cultured in RPMI medium supplemented with FBS and gentamycin.

### Sequence design and plasmid construction

An HIV-1 proviral plasmid containing the enhanced green fluorescent protein *(EGFP)* gene in the *Nef* position (NHG, GenBank: MF944225.1) was engineered to contain unique *BstEII* and *ClaI* restriction sites at nucleotide positions 6325 and 6771, respectively, within the *env* gene. DNA sequences comprising the coding region between the *BstEII* and *ClaI* restriction sites were designed to modulate the number and location of introduced CpG dinucleotides as well as the background mononucleotide frequency, without changing the encoded amino acids. For mutants in which the number of CpG dinucleotides was changed, the CpG dinucleotides were introduced at the codon boundaries by replacing the wobble position of the 5′ codon by a cytidine, or by substituting the wobble position of alanine, threonine, proline and serine codons with a guanine. The sequences surrounding each CpG dinucleotide was retained as in the HIV-1 NHG wildtype sequence. For mutants with variable spacing between each CpG dinucleotide and in mutants with different locations across the *Env* gene, a similar approach was adopted in which the wobble position of codons was modified to introduce a CpG at codon boundaries or within codons. Similarly, for modified wobble positions, the surrounding sequences were maintained as they appear in the wildtype sequences, with an exception: in some spacing mutants, four serine codons were substituted (AGT/AGC-->TCG). Finally, mutants with modified mononucleotide composition were designed by maintaining the position of the 14 CpG dinucleotides in the CG-14 virus and replacing all other codons with codons that contained the desired nucleotide. For example, in A+ mutants, leucine codons (CUU) were replaced with adenine-containing leucine codons (CUA), arginine codons (AGG) with adenine-containing codons (AGA) and so on. In all cases, no additional CpG dinucleotides were introduced. All sequences were designed with in-house built scripts and checked for inadvertent introduction of splice sites using MaxEntScan^38^. Synthetic DNA sequences encoding the modified sequences were purchased (Twist Bioscience) inserted into the HIV-1 NHG *BstEII* and *ClaI* modified proviral plasmid using standard cloning procedures.

Plasmids encoding the enterovirus A71 strain 41^24^ were used as a basis for the construction of the EV-A71 mutants. DNA sequences encoding the enteroviral polyprotein region between D1270 and R1586 were designed to modulate the number and location of CpG dinucleotides as well as the surrounding adenine content, without changing the amino acid sequence, as described above. Synthetic DNA sequences encoding the modified sequences were purchased (Twist Bioscience) and inserted into the EV-A71 genome plasmid using the *BstEII* and *SacII* restriction sites. A reporter EV-A71 encoding NanoLuc luciferase was generated as previously described^24^ by inserting the NanoLuc gene followed by a 2A cleavage site at the N terminus of the EVA-71 polyprotein. Mouse-adapted versions of EV-A71 wild type and EV-A71/CG-48/A+ were generated by inserting the mouse-adaptive substitutions described previously^25^. These mutations are K149I in VP2, plus Q145E and K244E in VP1. Additionally, to improve the replication of these viruses in human cell lines, the previously described substitution (H37K in VP1)^27^ was also introduced.

### Virus production

To produce HIV-1 mutant and wildtype virus stocks, HEK293T ZAP^−/−^ TRIM25^−/−^ cells were transfected with HIV-1 NHG proviral plasmids along with a plasmid encoding the vesicular stomatitis virus glycoprotein (VSV-G). The next day, cell culture media were replaced and at 48 h post-transfection, supernatants were collected, clarified by centrifugation (10 min, 2,000 × *g*) and filtered through a 0.22 µm filter. Collected viruses were concentrated using Lenti-X concentrator (Clontech) according to the manufacturer’s guidelines and resuspended in serum-free DMEM.

EV-A71 wildtype and mutant viruses were generated as described previously^24^. Briefly, viral plasmids were linearized with *MluI* restriction enzyme and column purified. Linearized DNA was then used to generate viral RNA using the T7 RiboMAX Express large-scale RNA production system according to the manufacturer’s guidelines. Viral RNA was then transfected in ZAP-deficient RD cells using the TransIT-mRNA transfection kit. After overnight incubation, media were replaced and cells were monitored for cytopathic effect. When cytopathic effect was observed in ~80% of cells, supernatants were collected and filtered through a 0.1 µm filter. Virus stocks were passaged once in ZAP-deficient RD cells. All virus stocks were stored at −80 °C before use.

### HIV-1 replication assays

For spreading infections, 1.5 × 10^5^ MT4 cells were infected with 400 infectious units of VSV-G pseudotyped HIV-1 NHG in a total of 2 ml of complete RPMI. Each day after infection, cells were resuspended, 100 µl of cell suspension was collected and fixed in 4% paraformaldehyde, and cultures were replenished with 100 µl of fresh RPMI. The percentage of GFP-positive cells was determined using flow cytometry and calculated using FlowJo. For the long-term virus passage experiments, 7.5 × 10^5^ MT4 cells were infected with 2,000 infectious units of the CG-43 or CG-15 HIV-1 NHG mutants. Every 2 d, cells were resuspended, 100 µl of the cell suspension was fixed in 4% paraformaldehyde and the percentage of GFP-positive cells was measured using a flow cytometer. When the percentage of GFP-positive cells was greater than 85%, supernatants were collected, filtered through a 0.22 µm filter and used as inoculum to infect fresh MT4 cells. Viral RNA was isolated from an aliquot of the passaged supernatant using TRIzol, reverse transcribed using the SuperScript III first-strand synthesis system, and an *env* fragment was amplified using the following primers: 5′-ACAGAAAAATTGTGGGTCACCGTCTATTATGGG-3′ and 5′-GCTGGTAGTATCATTATCGATTGGTATTATATCAAG-3′. Mutations identified in revertant viruses (S115 G6565A, P118 G6574A) were then introduced into the HIV-1 NHG CG-15 construct by site-directed mutagenesis. Virus stocks were generated as described above and their replication assessed in a spreading infection assay.

### EVA-71 replication assays

HeLa cells were infected at a multiplicity of infection (MOI) of 0.02 for 1 h at 37 °C. Cells were then washed twice in PBS and incubated in complete DMEM at 37 °C. At the indicated timepoints, 100 µl of the culture supernatant was collected and incubated with 25 µl of 5x concentrated passive lysis buffer (Promega) at room temperature for 5 min. NanoLuc luciferase activity was measured using the Nano-Glo luciferase assay system (Promega) according to the manufacturer’s guidelines. For long-term virus passage experiments, cells were infected as above and at 4 d post infection, supernatants were collected, clarified by centrifugation (10 min, 2,000 × *g*) and filtered through a 0.22 µm filter. Collected virus was diluted and used to re-infect cells at MOI = 0.02. Median tissue culture infectious doses (TCID_50_) were determined using a cytopathic effect readout and calculated as previously described^39^ using RD ZAP^−/−^ target cells.

### Immunoblotting

Cells were lysed in NuPAGE LDS sample buffer (Invitrogen) supplemented with β-mercaptoethanol. Samples were then heated at 72 °C for 20 min and sonicated for 15 s. Protein samples were resolved onto NuPAGE 4–12%, Bis-Tris protein gels (Invitrogen), transferred to nitrocellulose membranes and blocked with Intercept blocking buffer (Li-Cor) and incubated with the following antibodies: anti-ZC3HAV1 (rabbit polyclonal antibody, 16820-1-AP, Proteintech) was used at 1:5,000 dilution in PBS supplemented with Tween20 in human MT4 cell line samples, anti-ZC3HAV1 (rabbit polyclonal antibody, abx124715, Abbexa) was used at 1:300 dilution in 5% milk in PBS-Tween20 in mouse peripheral blood mononuclear cell samples and anti-α-tubulin (mouse monoclonal antibody, T5168, Sigma). After overnight incubation at 4 °C, membranes were washed in PBS-Tween20, blotted with horseradish peroxidase-conjugated secondary antibodies and developed using a C-Digit chemiluminescent western blot scanner.

### CLIP-seq

All CLIP-seq experiments were performed as described previously^13^ with the following modifications. HEK293T ZAP^−/−^ TRIM25^−/−^ cells were transfected with a proviral plasmid and a plasmid encoding the long isoform of ZAP (ZAP-L) and three consecutive C-terminal hemagglutinin (HA) epitope tags. The next day, media were replaced and 4-thiouridine was added to the culture for 16 h. Cells were then washed with cold PBS and exposed to UV radiation. ZAP:RNA complexes were isolated by immunoprecipitation with an anti-HA antibody and RNA was ligated to a fluorescently labelled 3′ adapter. RNA was isolated and ligated to a 5′ adapter and reverse transcribed using the SuperScript IV first-strand synthesis system (Invitrogen). The resulting complementary DNA library was amplified using Illumina primers and sequenced using a NovaSeq sequencer (Rockefeller Genomics Resource Center). Reads were processed as described previously^4^ and aligned against the HIV-1 NHG genome as indicated.

### Mouse experiments and generation of ZAP-knockout mice

All mice used in this study were derived from the C57BL/6 line or the C57BL/6J *Ifnar1*^−/−^ knockout line (MMRRC, 32045)^40^. Mice of both sexes were used, except for the passive protective experiments in which females were specifically used (to generate immunized dams). All animal experiments were conducted according to The Rockefeller University Institutional Animal Care and Use Committee.

ZAP-knockout C57BL/6 mice were generated by the CRISPR and Genome Editing Resource Center at The Rockefeller University by zygote injection of Cas9 complexed with a guide RNA targeting exon 1 of mouse *ZC3HAV1* gene (ZAP-B guide sequence: 5′-AGTACTTGCGACGGCAGACGCGG-3′). The resulting pups were tailed and genomic DNA was extracted. Indels were determined by sequencing of the PCR products obtained using the following primers: ZAP-SA-F1 5′-GGGGTCTAACTTCACAGGAGT-3′ and ZAP-SA-R1 5′-CCTCACGTCTAGCCTGGAAC-3′. Two DNA lesions were identified, one of them (+2 insertion) was purified by breeding to homozygosity. ZAP^−/−^ IFNAR^−/−^ mice were generated by crossing homozygous ZAP-knockout mice with C57BL/6^IFNAR1−/−^ mice (B6.129S2-*Ifnar1*^tm1Agt^/Mmjax) purchased from Jackson Laboratories.

### Mouse infections with mEV71

To assess replication and pathogenicity of mEV-A71 and mutants thereof, 1-day-old suckling mice were infected by intraperitoneal injection with 1 × 10^5^ TCID_50_ of mouse-adapted mEV-A71 (wildtype) or mEV-A71/CG-48/A+. Pups were monitored daily for symptoms for a total period of 20 d. A clinical score was measured as described previously^26^ with the following modifications: 0, healthy; 1, weak/lethargic; 2, one-limb paralysis; 3, two-limb paralysis; 4, dead/moribund/euthanized. When two-limb paralysis was observed for a period of 48 h without recovery, affected mice were humanely euthanized. For the measurement of viral RNA, mice were euthanized at 6 d post infection. Skeletal muscle from rear limbs were collected and stored in RNAlater solution (Thermo) before processing. Organs were thawed and homogenized in TRizol LS reagent according to the manufacturer’s guidelines. Isolated RNA was reverse transcribed using the SuperScript III first-strand synthesis system (Invitrogen) and viral RNA molecules were quantified by quantitative RT-PCR using *Taq*Man gene expression master mix and the following oligonucleotides: probe EV-A71: 5′-6FAM- ATTCCAAAAGAAAGCACTATCCAGTCAGC-MGBNFQ-3′, forward primer: 5′-GAACCTCGTCTGGGAAGATAGCTCCC-3′ and reverse primer: 5′-TCGCCGGGCTCAGAGTGGCCT-3′.

For the passive protection experiments, female ZAP^+/+^*Ifnar1*^−/−^ mice that were previously infected with mEV-A71/CG-48/A+ virus as well as mock-treated females at 6-weeks of age were mated with naïve *Ifnar*^−/−^ males. The resulting offspring were infected with mEV-A71 wild type via the intraperitoneal route as described above. Infected mice were monitored daily until weaning age as described above.

### EV-A71 neutralizing antibody assays

Blood samples from mice inoculated with mEV71/CG-48/A+ were collected at 4 and 6 weeks after infection. Plasma was heat-inactivated as described previously^41^, serially diluted and incubated with EV-A71 NanoLuc reporter virus for 1 h at 37 °C. Viruses were then used to infect HEK293T cells and incubated for a further 48 h at 37 °C. Infected cells were then lysed with passive lysis buffer (Promega) and nano luciferase activity was measured as described above. NT_50_ titres were calculated in GraphPad Prism using non-linear regression (least squares regression without weighting).

### Statistical analysis

All statistical analyses were performed using GraphPad Prism 9. Spreading infection data were plotted as mean ± s.d. For comparison between virus mutants and presence of ZAP, two-way analysis of variance (ANOVA) was used with Šídák’s multiple comparisons test. Quantified viral RNA and luciferase activity were plotted as mean ± s.d. For the plasma neutralization data, both mean and SEM were plotted. We chose the indicated sample size for our animal experiments on the basis of our previous experiments using C57BL/6 mice in infection models^41^. To assess statistical significance in the clinical scores of infected mice, we performed two-way ANOVA, while differences in probability of survival were assessed using the Mantel-Cox test. Details of statistical tests, sample sizes as well as *P* values are described for each experiment in the respective figure legends.

### Reporting summary

Further information on research design is available in the Nature Research Reporting Summary linked to this article.

## Supplementary information

Reporting Summary

## Data Availability

The data that support the findings of this study are available in the accompanying Source Data files. The NHG HIV-1 genome sequence used in this study can be accessed through the NCBI nucleotide database using the accession code MF944225.1. Unprocessed raw data from CLIP-Seq experiments can be accessed through the NCBI Gene Expression Omnibus database using the accession code GSE208611.

## Source data

Source Data Fig. 1 Unprocessed western blots.
Source Data Fig. 1 Statistical source data.
Source Data Fig. 2 Statistical source data.
Source Data Fig. 3 Statistical source data.
Source Data Fig. 4 Statistical source data.
Source Data Fig. 5 Statistical source data.
Source Data Extended Data Fig. 1 Unprocessed western blots.
Source Data Extended Data Fig. 1 Statistical source data.
Source Data Extended Data Fig. 2 Statistical source data.
Source Data Extended Data Fig. 3 Statistical source data.
Source Data Extended Data Fig. 4 Statistical source data.
Source Data Extended Data Fig. 5 Statistical source data.
Source Data Extended Data Fig. 6 Statistical source data.
Source Data Extended Data Fig. 6 Unprocessed western blots.
Source Data Extended Data Fig. 7 Statistical source data.
Source Data Extended Data Fig. 8 Statistical source data.

## Code availability

Code used to map and calculate read counts from CLIP-seq experiments was obtained from http://hannonlab.cshl.edu/fastx_toolkit/ and can be accessed from GitHub under https://github.com/agordon/fastx_toolkit.
