# Supplementary material for: Rational attenuation of RNA viruses with zinc finger antiviral protein
Source: Nat Microbiol. 2022 Sep 8;7(10):1558–67. doi: 10.1038/s41564-022-01223-8 (PMC9519448; doi:10.1038/s41564-022-01223-8)
Supplement: Supplementary file 1 — Reporting Summary [file 41564_2022_1223_MOESM1_ESM.pdf]

nature portfolio

Corresponding author(s): Paul D. Bieniasz

Last updated by author(s): Jul 27, 2022

Reporting Summary

Nature Portfolio wishes to improve the reproducibility of the work that we publish. This form provides structure for consistency and transparency in reporting. For further information on Nature Portfolio policies, see our Editorial Policies and the Editorial Policy Checklist.

Statistics

For all statistical analyses, confirm that the following items are present in the figure legend, table legend, main text, or Methods section.

n/a	Confirmed	
☐	☒	The exact sample size (*n*) for each experimental group/condition, given as a discrete number and unit of measurement
☐	☒	A statement on whether measurements were taken from distinct samples or whether the same sample was measured repeatedly
☐	☒	The statistical test(s) used AND whether they are one- or two-sided<br>*Only common tests should be described solely by name; describe more complex techniques in the Methods section.*
☒	☐	A description of all covariates tested
☒	☐	A description of any assumptions or corrections, such as tests of normality and adjustment for multiple comparisons
☐	☒	A full description of the statistical parameters including central tendency (e.g. means) or other basic estimates (e.g. regression coefficient) AND variation (e.g. standard deviation) or associated estimates of uncertainty (e.g. confidence intervals)
☐	☒	For null hypothesis testing, the test statistic (e.g. *F*, *t*, *r*) with confidence intervals, effect sizes, degrees of freedom and *P* value noted<br>*Give P values as exact values whenever suitable.*
☒	☐	For Bayesian analysis, information on the choice of priors and Markov chain Monte Carlo settings
☒	☐	For hierarchical and complex designs, identification of the appropriate level for tests and full reporting of outcomes
☒	☐	Estimates of effect sizes (e.g. Cohen's *d*, Pearson's *r*), indicating how they were calculated

Our web collection on statistics for biologists contains articles on many of the points above.

Software and code

Policy information about availability of computer code

Data collection	CLIP-Seq data were processed and analyzed using python scripts as part of the FASTX-Toolkit version-0.0.14 (available here: Code used to map and calculate read counts from CLIP-Seq experiments is available from http://hannonlab.cshl.edu/fastx_toolkit/ and can be cloned from GitHub under https://github.com/agordon/fastx_toolkit).
Data analysis	Flow cytometry data were analyzed using FlowJo version 10.8.0. All other data were plotted and analyzed using GraphPad Prism version 9.2.0. Statistical test were performed using GraphPad Prism version 9.2.0. Analysis of potential splicing sites in recoded sequences was performed using MaxEntScan (available here: http://hollywood.mit.edu/burgelab/maxent/Xmaxentscan_scoreseq.html)

For manuscripts utilizing custom algorithms or software that are central to the research but not yet described in published literature, software must be made available to editors and reviewers. We strongly encourage code deposition in a community repository (e.g. GitHub). See the Nature Portfolio guidelines for submitting code & software for further information.

Data

Policy information about availability of data

All manuscripts must include a data availability statement. This statement should provide the following information, where applicable:

- Accession codes, unique identifiers, or web links for publicly available datasets
- A description of any restrictions on data availability
- For clinical datasets or third party data, please ensure that the statement adheres to our policy

The data that support the findings of this study are available in the Source Data files. Sequencing data resulting from CLIP-Seq experiments have been deposited in the NCBI GEO database and can be accessed using the accession code GSE208611. The NHG HIV-1 genome we used in this study can be accessed through the NCBI Nucleotide database using the accession code MF944225.1.

Human research participants

Policy information about studies involving human research participants and Sex and Gender in Research.

Reporting on sex and gender	N/A
Population characteristics	N/A
Recruitment	N/A
Ethics oversight	N/A

Note that full information on the approval of the study protocol must also be provided in the manuscript.

Field-specific reporting

Please select the one below that is the best fit for your research. If you are not sure, read the appropriate sections before making your selection.

☒ Life sciences ☐ Behavioural & social sciences ☐ Ecological, evolutionary & environmental sciences

For a reference copy of the document with all sections, see nature.com/documents/nr-reporting-summary-flat.pdf

Life sciences study design

All studies must disclose on these points even when the disclosure is negative.

Sample size	Sample size was calculated based on previous experiments and the authors' experience. For animal experiments, animal group size was determined based on Tee et al. 2019 Plos Pathogens (PMID: 31730673), who used similar infection conditions with EV-A71.
Data exclusions	No data was excluded from this study with the exception of some CLIP experiments with read counts that were too low to be reliable.
Replication	All experiments presented in this manuscript were reliably replicated, at least 3 times, and presented equivalent results. CLIP experiments were performed twice, since these are inherently complex experiments with frequent reagent deterioration. Both experiments yielded remarkably similar results with very high read counts.
Randomization	All mice were infected at the same age and with the same infectious dose. Littermates of both sexes were included in the same experimental groups. Mice litters were randomly chosen to be infected with wildtype or mutant viruses as they became available from ongoing matings. Female mice that survived infection were then paired with non-exposed males; their offspring was challenged with wiltype viruses as mice litters become available without deliberate selection. All other experiments that did not involve mice were not subject to randomization since experimental units measurements were performed by machines and not subject to operator's bias.
Blinding	No blinding was used in this study since most of the quantitative data presented were measured by a machine. In infection experiments in mice that required the attribution of a previously published clinical score, we based scores on the observation of clear and obvious symptoms including death of the animal, the complete paralysis of 1 or 2 limbs, and hunched position adopted by some mice. All infections in mice resulted in a binary survival score (dead or alive) that could not be subject to interpretation by the operator.

Reporting for specific materials, systems and methods

We require information from authors about some types of materials, experimental systems and methods used in many studies. Here, indicate whether each material, system or method listed is relevant to your study. If you are not sure if a list item applies to your research, read the appropriate section before selecting a response.

Materials & experimental systems

n/a	Involved in the study
☐	☒ Antibodies
☐	☒ Eukaryotic cell lines
☒	☐ Palaeontology and archaeology
☐	☒ Animals and other organisms
☒	☐ Clinical data
☒	☐ Dual use research of concern

Methods

n/a	Involved in the study
☒	☐ ChIP-seq
☒	☐ Flow cytometry
☒	☐ MRI-based neuroimaging

Antibodies

Antibodies used	Anti-ZC3HAV1 Rabbit Polyclonal Antibody (Anti-ZAP), ProteinTech, 16820-1-AP, Lot# 00046982. Used in western blotting at 1:5000 dilution.<br>Anti-α-Tubulin Mouse Monoclonal Antibody, Millipore-Sigma, T5168, Batch# 0000089494. Used in western blotting at 1:10,000 dilution.<br>Anti-ZC3HAV1 Rabbit Polyclonal Antibody, Abbexa, abx124715, Lot#A2002958K. Used in western blotting at 1:300 dilution.<br>Anti-HA epitope tag Rabbit Monoclonal Antibody, clone 600-401-384, Rockland. Used in western blotting at 1:5000.<br>Anti-HIV-1-Env (Gp160/Gp120), Goat Polyclonal Antibody, clone 12-6205-1, American Research Products. Used in western blotting at 1:1000 dilution.
Validation	Anti-ZC3HAV1 (ProteinTech) antibody was validated for western blotting in siRNA and gRNA experiments targeting ZAP in several human cells (PMID: 28953888).<br>Anti-α-Tubulin antibody was validated by manufacturer (Sigma's Enhanced Validation antibodies, https://www.sigmaaldrich.com/US/en/product/sigma/t5168) and by other research groups against protein extracts from human cells (PMID: 31434934) and multiple mouse organs (PMID: 23748901).<br>Anti-ZC3HAV1 (Abbexa) antibody was validated by manufacturer for western blotting by using mouse lung and thymus protein extracts (https://www.abbexa.com/zc3hav1-antibody) and in this study (Extended Figure 6) using mouse PBMCs from ZAP+/+ and ZAP-/- mouselines.<br>Anti-HA (Rockland) antibody was validated for western blotting by the manufacturer (https://www.rockland.com/categories/primary-antibodies/ha-epitope-tag-antibody-600-401-384/?gclid=Cj0KCQjwof6WBhD4ARIsAOi65aganITbyl6riiH7x99D_XsM2C4i6lTSYAa_TN1j3opEyPl32mHGnGQaAlUeEALw_wcB) as well as in this study by analyzing cell extracts containing HA-tagged proteins.<br>Anti-HIV-1-Env was validated for western blotting previously (PMID: 33901262) and in this study by analyzing protein extracts from cells infected with HIV-1.

Eukaryotic cell lines

Policy information about cell lines and Sex and Gender in Research

Cell line source(s)	Human Embryonic Kidney 293T (HEK293T) and HeLa cells were obtained from ATCC. MT4 human lymphocyte cell line was obtained from the NIH AIDS Reagent repository.
Authentication	Cells were authenticated by microscopic inspection.
Mycoplasma contamination	Cell lines were not tested for the presence of mycoplasma.
Commonly misidentified lines<br>(See ICLAC register)	No commonly misidentified lines were used in this study.

Animals and other research organisms

Policy information about studies involving animals; ARRIVE guidelines recommended for reporting animal research, and Sex and Gender in Research

Laboratory animals	House mouse (Mus musculus), C57BL/6J, used in experiments and/or colony maintenance between 1-day old and 9-months of age. A transgenic ZC3HAV1 knockout line (homozygous ZAP-knockout) was derived from C57BL/6J. We also used a C57BL/6J Ifnar1-/- knockout line (MMRRC #32045). All mice were housed in standard housing conditions (12h light-dark cycles, room temperature of 65-75°F and 40-60% humidity). Mice had unrestricted access to the food and water.
Wild animals	We did not use any wild animals.
Reporting on sex	Sex was not considered in this study. We used mice of both sexes in disease progression experiments and females for maternal antibody protection experiments.
Field-collected samples	This study did not involve field-collected samples.
Ethics oversight	Mouse handling and experimental protocol (18047-H) was approved by institutional animal care and use committee (IACUC) protocol

Ethics oversight	of the Rockefeller University.

Note that full information on the approval of the study protocol must also be provided in the manuscript.
