# Supplementary figures and images for: Rational attenuation of RNA viruses with zinc finger antiviral protein
Source: Nat Microbiol. 2022 Sep 8;7(10):1558–67. doi: 10.1038/s41564-022-01223-8 (PMC9519448; doi:10.1038/s41564-022-01223-8)

Uncropped western blots in Figure 1b

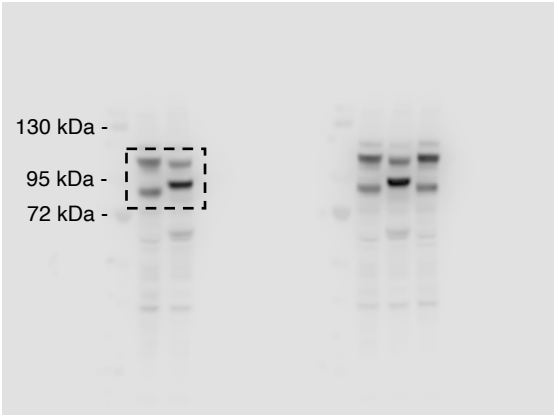

Anti-ZAP

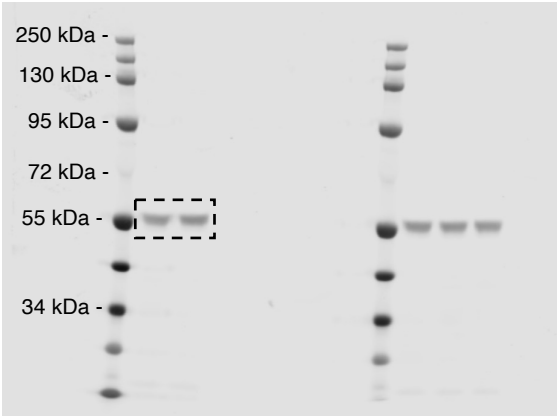

Anti-Tubulin

Supplement: Source Data Fig. 1 — Unprocessed western blots. [file 41564_2022_1223_MOESM2_ESM.pdf]

Uncropped western blots in Extended Figure 1c

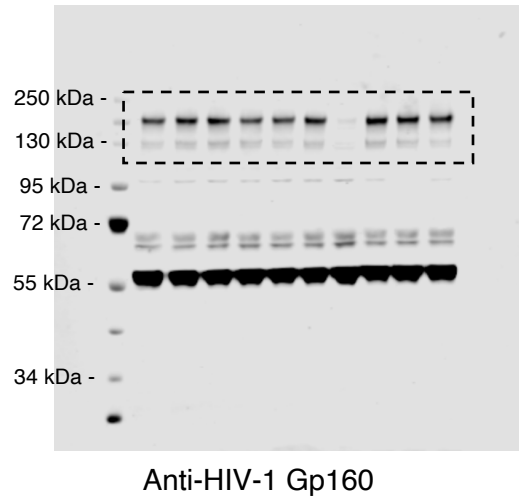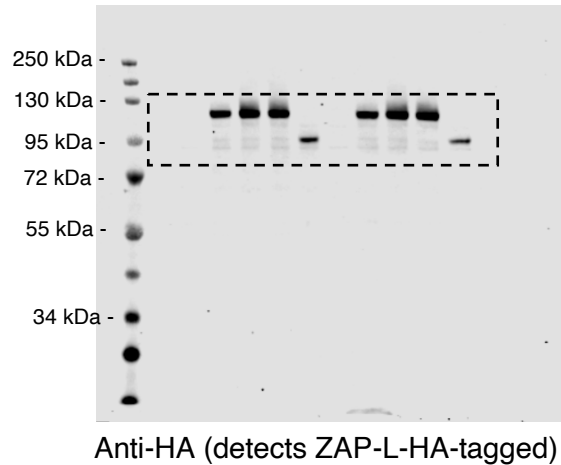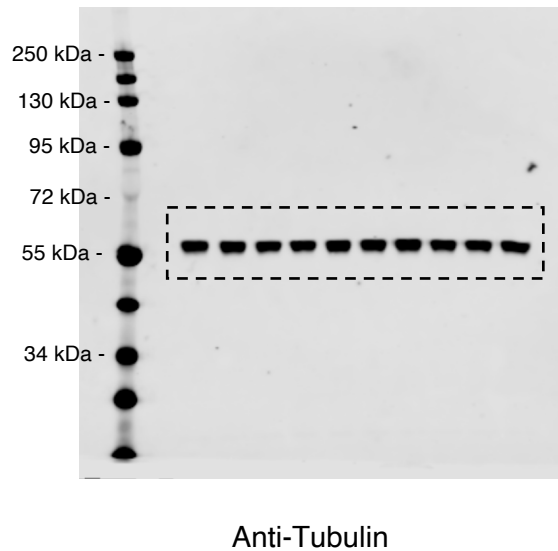

Supplement: Source Data Extended Data Fig. 1 — Unprocessed western blots. [file 41564_2022_1223_MOESM8_ESM.pdf]

Uncropped western blots in Extended Figure 6b

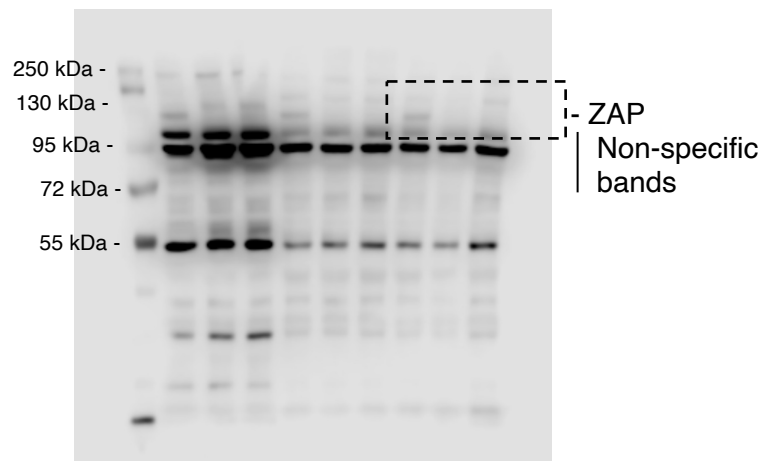

Anti-ZAP

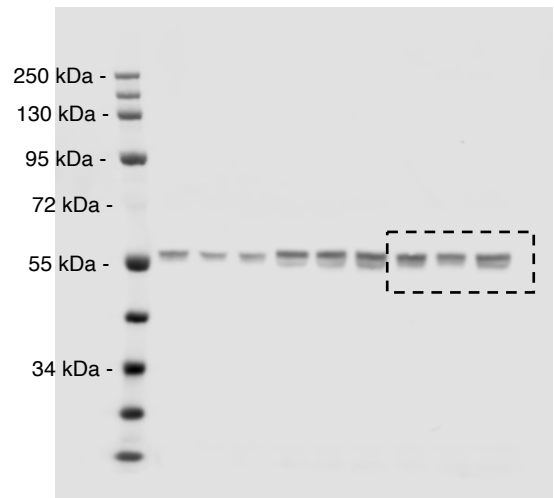

Anti-Tubulin

Supplement: Source Data Extended Data Fig. 6 — Unprocessed western blots. [file 41564_2022_1223_MOESM15_ESM.pdf]
